# Supplementary material for: The causal relationship between the human gut microbiota and pyogenic arthritis: a Mendelian randomization study
Source: Front Cell Infect Microbiol. 2024 Nov 26;14:1452480. doi: 10.3389/fcimb.2024.1452480 (PMC11629706; doi:10.3389/fcimb.2024.1452480)
Supplement: Supplementary file 1 [file DataSheet1.zip › mendelian test/ebi-a-GCST90027628.csv_leave_one_out.pdf]

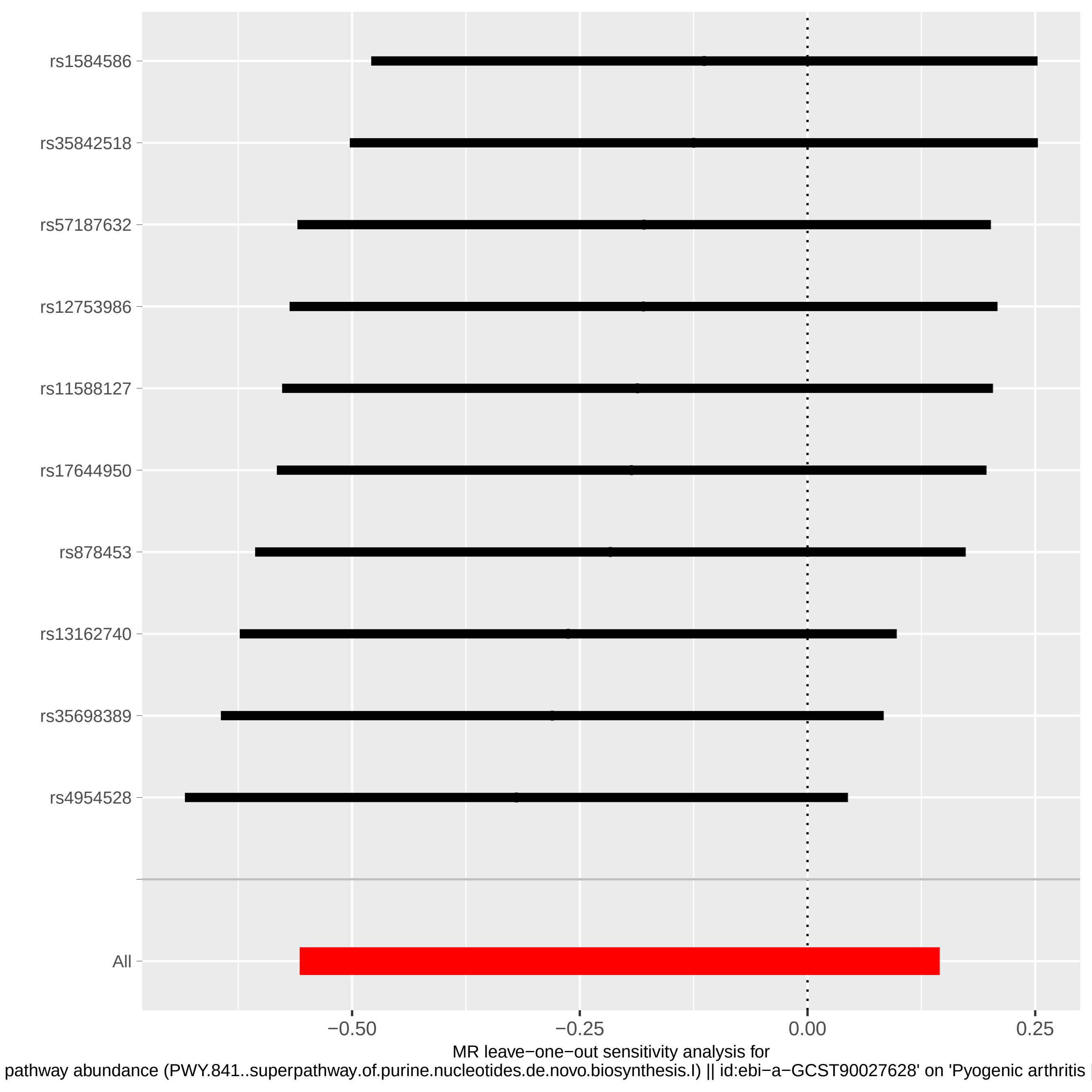
rs1584586
rs35842518
rs57187632
rs12753986
rs11588127
rs17644950
rs878453
rs13162740
rs35698389
rs4954528
All
−0.50
−0.25
0.00
0.25
MR leave−one−out sensitivity analysis for
pathway abundance (PWY.841..superpathway.of.purine.nucleotides.de.novo.biosynthesis.I) || id:ebi−a−GCST90027628' on 'Pyogenic arthritis
